# Pineal Gland Tumor but not Pinealoma: A Case Report

**DOI:** 10.7759/cureus.1576

**Published:** 2017-08-18

**Authors:** Syeda Naqvi, Chintan Rupareliya, Abdullah Shams, Maria Hameed, Zabeen Mahuwala, Pirthvi Raj Giyanwani

**Affiliations:** 1 Jinnah Postgraduate Medical Centre, Jinnah Sindh Medical University (SMC); 2 Department of Neurology, University of Missouri, Columbia, Missouri; 3 Internal Medicine, CMH Lahore Medical and Dental College; 4 Department of Neurology, University Of Kentucky College of Medicine; 5 Civil Hospital Karachi, Dow University of Health Sciences, Karachi, Pakistan

**Keywords:** pineal gland, tumor, headache, oligodendroglioma, visual disturbance

## Abstract

The pineal gland is a small pinecone-shaped and functionally endocrine structure located in the epithalamus region. Developmentally, the pineal gland is considered as a part of the epithalamus. It plays a role in the entrainment of the circadian rhythms of an organism by producing melatonin, a functionally important hormone. Lesions of the pineal region are rare compared to other parts of the brain. A lesion may be tumorous or non-tumorous in nature. The most common lesions are tumors that are pineal parenchymal tumors (PPT) in origin. Gliomas are the second most common tumors in the pineal region. We report a case of a high-grade oligodendroglioma, not commonly seen in the pineal region, in a 45-year-old male. The patient was suspected to have a mass in the pineal region on a computed tomography (CT) scan and histology confirmed the diagnosis of oligodendroglioma. This is a unique case because only five such cases have been reported so far.

## Introduction

The pineal gland is a small endocrine gland located in the epithalamus region. It produces melatonin, which helps in modulating the sleep cycle. Malignancies of the pineal gland arising from itself are very rare. The spectrum of tumors arising in this region is broad. Almost more than 17 heterogeneous tumors can arise in this region.

To our knowledge, only five cases of oligodendroglioma of the pineal region have been reported in the literature. This is the sixth case, which makes this incidence a unique presentation.

## Case presentation

A 45-year-old man presented to the outpatient department with complaints of a frontal headache for the last four months. His pain was 6/10 on the intensity scale. The headache usually occurred in the morning, was severe in intensity, was associated with dizziness, and usually responded to over-the-counter pain medication. For past four months, the patient reported visual disturbances in the form of blurry vision, but on thorough examination by the ophthalmology department, a peripheral visual defect was ruled out and magnetic resonance imaging (MRI) of the brain was advised.

**Figure 1:**
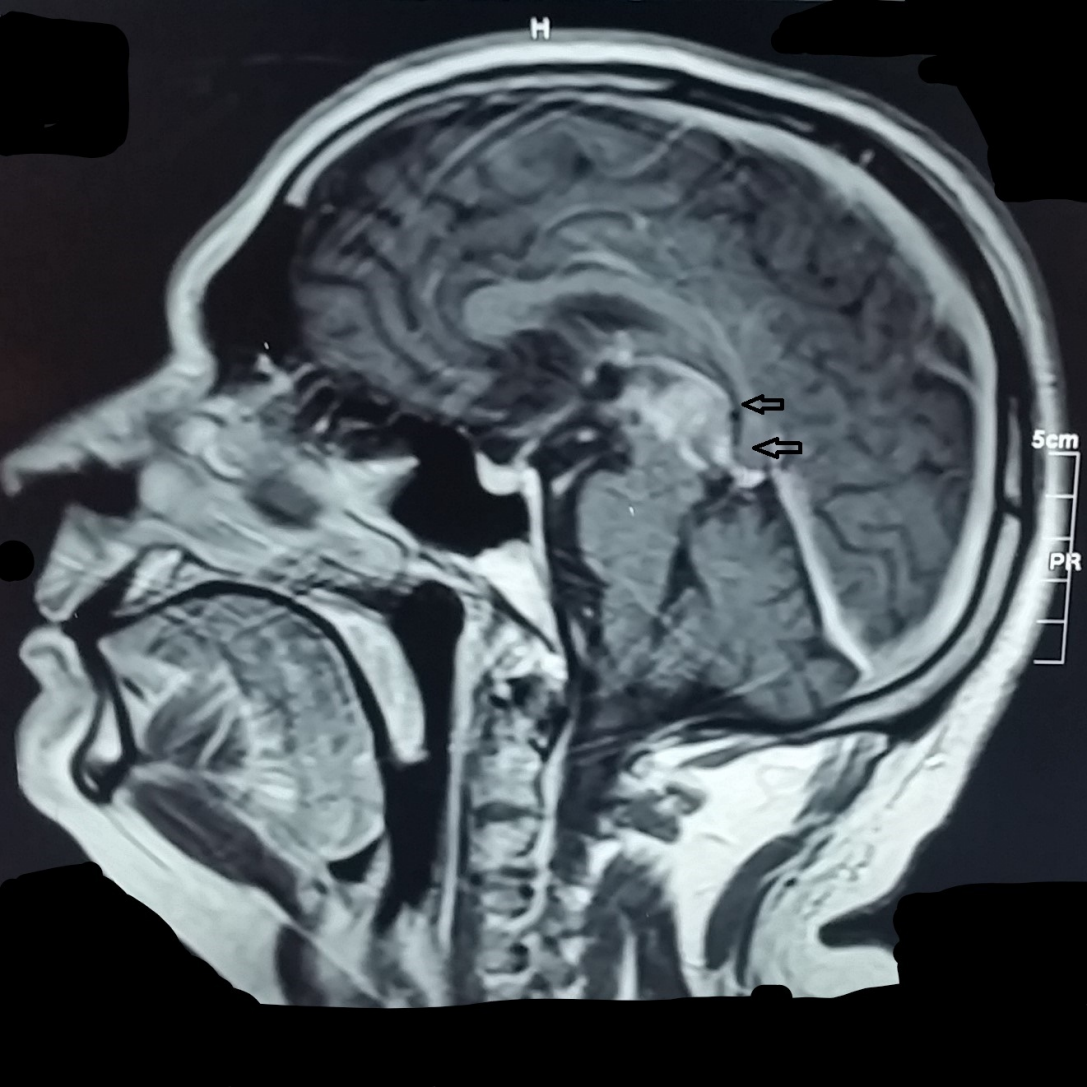
Tumor in the Pineal Region

**Figure 2:**
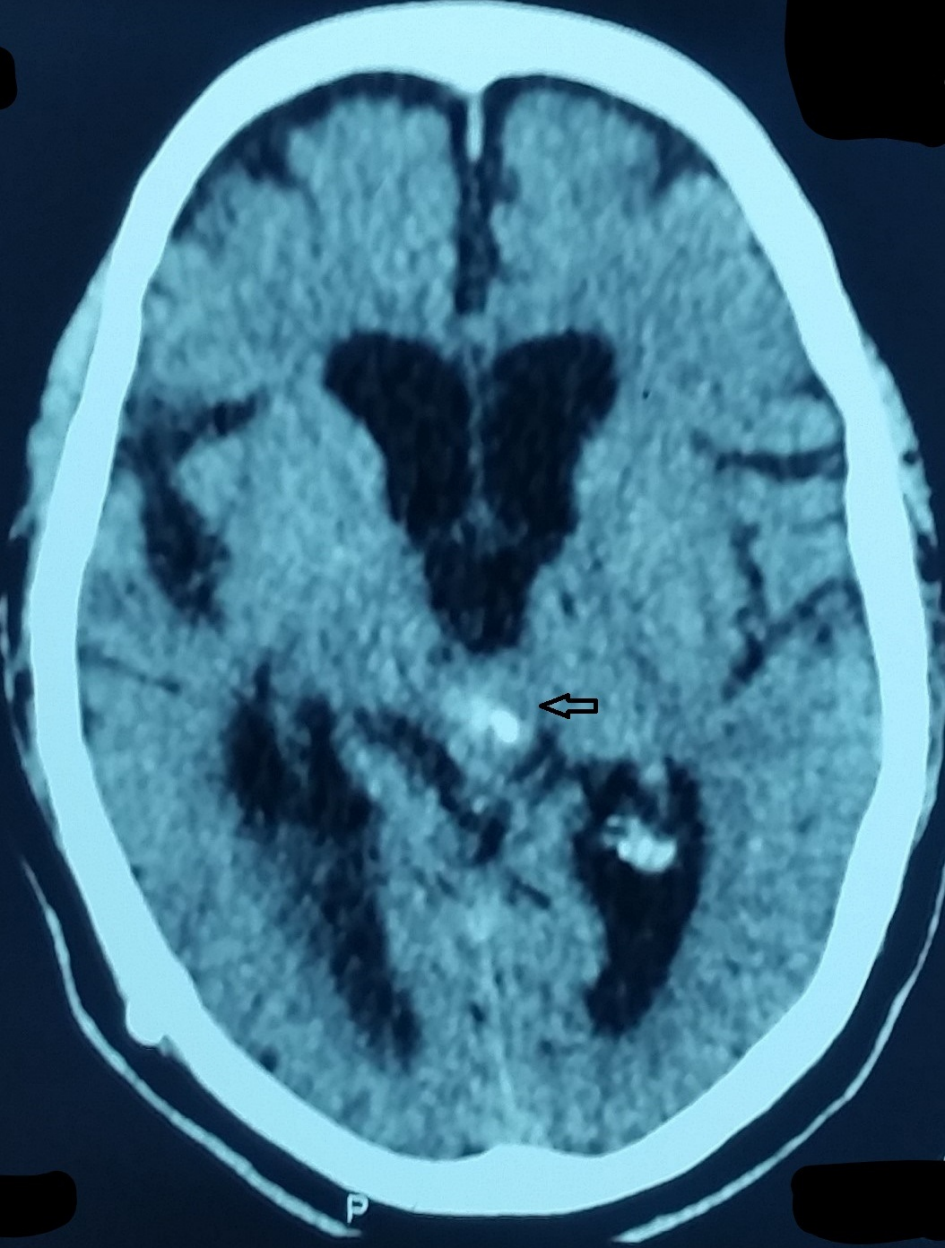
Pineal Region Mass on CT Scan CT - computed tomography.

The patient underwent a brain MRI and was referred to the medicine outpatient department where it was revealed that the patient had a mass in the pineal region as shown in Figure 1 and the CT scan in Figure 2.

His baselines including the complete blood count, basic metabolic panel, liver function test, urine detailed report and chest X-ray were within normal limits. The patient denied any family history of brain tumors. His past medical history included diabetes and hypertension for the last five years. Both were well controlled on losartan and Glucophage, respectively.

Neurological consult was sought and the patient was advised to get a brain biopsy done to channel the diagnosis into effective treatment options. The sample was processed and sent for histology. It showed a neoplastic lesion composed of polygonal cells against a fibrillary background. These neoplastic cells had a round nucleus exhibiting a moderate degree of pleomorphism and surrounded by a perinuclear halo. Significantly increased mitotic activity was appreciated. The immunohistochemical analysis came with positive CD 57, CD99, CD 10, diffuse positive for GFAP (glial fibrillary acidic protein), weak positivity for synaptophysin, and a high Ki-67 index (around 17 percent of cells). Cytokeratin and extractable nuclear antigen (ENA) immunohistochemical stains were also negative. Fluorescent insitu hybridization (FISH) performed on a small number of cells showed deletion of 1p and 19q.

In the light of all these findings, a diagnosis of oligodendroglioma was concluded. The patient was offered radiation and chemotherapy options like procarbazine, methyl-1-(2-chloroethyl)-1-nitrosourea (CCNU) and vincristine (also known as PCV therapy). After a detailed discussion, the patient and his family declined both options. Surgical management was also proposed but the patient deferred it.

## Discussion

Oligodendrogliomas, a type of glioma, are believed to originate from the oligodendrocytes of the brain or from a glial precursor cell. Overall, they constitute less than 20% of all intracranial tumors and less than 25% of gliomas [1]. Their prevalence is mostly among adults during midlife and rare in the pediatric population, occurring exclusively in the cerebral hemisphere predominantly in the male population with a male to female ratio of 1.28:1 [1-3]. Most cases have been reported in Caucasian, African American, and Hispanic populations. On diagnostic imaging, these tumors are mostly found in the temporal lobe in the pediatric population, while in adults they have been mostly reported in the frontal cortex region [1]. They occur at a median age of 30 and the most common presenting symptoms are headache (78%), seizures (43%), motor symptoms (38%), and to a lesser extent behavioral changes [4-5].The pathologic grade of the tumor has been an essential element in determining the progression of symptoms and survival rate. Patients with low grade or pediatric patients with good performance status at initial presentation had a better survival rate following treatment [4, 6].

**Figure 3:**
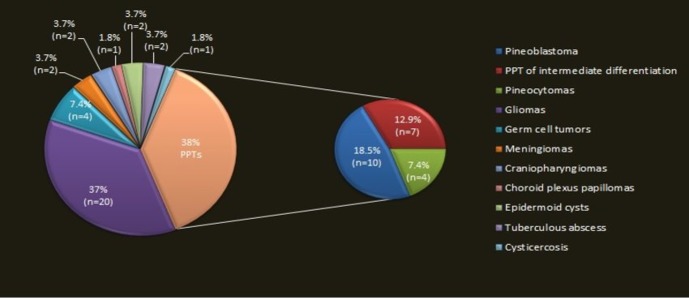
Pinealoma Subtypes on the Basis of Histology The smaller pie chart contains three subtypes of pineal parenchymal tumors (PPT).

The pineal region is a complex anatomical site sheltering the pineal gland surrounded by the quadrigeminal plate and the confluence of the internal cerebral veins forming the vein of Galen [7]. Although germinal cell tumors and pineal cell tumors form the majority of the histological tumors, more than 17 different tumors can arise in this location [8]. A pie chart distribution of tumor and non-tumor lesions of the pineal gland by the histological sub type based on an observational study of 12 years is shown in Figure 3 [2].

Gliomas are a very rare subtype of pineal region tumors; oligodendrogliomas of the pineal region, being a subtype of gliomas, further contribute to exclusively rare tumors with very few reported cases reported till date [5, 9].

As stated previously, the grade of the tumor determines the course of the disease and treatment, with the overall five-year progression-free survival rate being around 79% for low-grade tumors as compared to 39% for high-grade tumors [3]. Pineal tumors and especially germinal tumors are chemosensitive and radiosensitive; care of these tumors is multidisciplinary involving surgery, chemotherapy, and radiotherapy [3-4, 9]. Our patient was offered the treatment but he and his family members refused to agree to surgical resection or any form of chemo and radiotherapy. However, they did agree to frequent follow-ups after proper counseling as a part of palliative treatment.

To our knowledge, only five cases of oligodendroglioma of the pineal region have been reported in the literature. This is the sixth case, which makes this incidence a unique presentation. The findings of all previous five cases and the current case are compiled in Table 1. The details of the five cases are mentioned after the literature review of the corresponding case [2, 10].

**Table 1:** Cases of Oligodendroglioma in the Pineal Gland

Age, Sex	Month, Year of reporting	Presenting Symptoms	Histological Diagnosis	Treatment Given
30, Male	January, 2006	Not mentioned	Anaplastic OD	Not mentioned
35, Female	January, 2006	Not mentioned	Anaplastic OD	Not mentioned
59, Female	September, 2006	Headache + intermittent confusion + memory disturbance	Anaplastic OD	Surgical resection + chemo- & radiotherapy
37, Female	September, 2010	Diplopia	Low-grade OD	Surgical resection
22, Female	February, 2015	Headache of increasing severity for one month	Low-grade OD	Surgical resection
45, Male (Current Case)	July, 2017 (Current Case)	Frontal headache + transient visual obscuration (Current Case)	High-grade OD (Current Case)	Chemo- & radiotherapy (Patient denied surgical resection) (Current Case)

## Conclusions

We present this case of oligodendroglioma in the pineal gland as it is the sixth case reported till now. This case will serve to remind physicians about its occurrence and presentation. A headache as the initial presentation of many illnesses should be given due consideration and one should rule out every possible cause. This, in turn, would help us to design a management layout for such an exceedingly rare tumor.
